# Reply to Borgia et al. Comment on “Marasca et al. Teledermatology and Inflammatory Skin Conditions during COVID-19 Era: New Perspectives and Applications. *J. Clin. Med.* 2022, *11*, 1511”

**DOI:** 10.3390/jcm11144244

**Published:** 2022-07-21

**Authors:** Luca Potestio, Luigi Fornaro, Fabrizio Martora, Vincenzo Picone, Gabriella Fabbrocini, Claudio Marasca

**Affiliations:** Section of Dermatology, Department of Clinical Medicine and Surgery, University of Naples Federico II, Via Pansini 5, 80131 Napoli, Italy; potestioluca@gmail.com (L.P.); fornaroluigi95@gmail.com (L.F.); fabriziomartora92@libero.it (F.M.); vince.picone95@gmail.com (V.P.); gafabbro@unina.it (G.F.)

Telemedicine can be defined as a modern technology supporting health care from a distance [1].

We have read the interesting article recently published by Borgia et al. [2] commenting on our review analyzing perspectives and applications on the use of teledermatology in inflammatory skin conditions during the COVID-19 era [3]. Borgia et al. highlighted the utility of telemedicine in overcoming geographical distances and time limits, enabling people to access health care without leaving home [2]. The authors showed the need for an app for smartphones and personal computers that requires users to upload photographs to assess the severity of disease, allowing e-lab and e-drug prescriptions [2]. Similarly, several severity score indexes should be revisited in order to make them applicable to telemedicine [2]. However, the authors pointed out the need for a high-quality camera, the capacity for using devices and internet access as the main limitations of telemedicine. Moreover, the physician–patient relationship should not be ignored [2]. Thus, Borgia et al. suggested the association of televisits with face-to-face consultations in order to offer continuous assistance without harming the physician–patient relationship [2].

In our review, we underlined how COVID-19 revolutionized the management of several dermatologic conditions [4]. Indeed, COVID-19 restriction measures forced clinicians to limit their outpatient visits to serious cases, referring to telemedicine for chronic disease management. Several options were adopted to contain the spread of the infection, offering patients a high standard of care [5]. In contrast to Borgia et al., we highlighted several strategies of telemedicine, such as email, phone or video calls, and WhatsApp and Facebook support groups. We agree with the authors’ proposal of the need for an application that can standardize the use of teledermatology, and of the requirement for new severity indexes to classify disease severity using teleconsultation. Herein, we aim to underline the potential role of teledermatology in the near future.

Nowadays, the reduction in COVID-19 restriction measures and the lifting of social distancing requirements have led to an increase in the number of face-to-face consultations, reducing the need for synchronous and asynchronous evaluations [6]. Certainly, teledermatology reduces logistical barriers, time constraints and costs, and televisits were shown to be easily accessible, safe and effective. In our opinion, patients undergoing biological treatment for chronic inflammatory skin diseases, such as psoriasis, atopic dermatitis and hidradenitis suppurativa, or with a controlled disease may benefit the most from teledermatology [7]. Moreover, teledermatology remains a useful tool to monitor and guide patients in their disease treatment, and other dermatological conditions, such as skin cancer, may also benefit from teledermatology [8].

In addition, teledermatology will continue to be the main strategy in subjects who need disease assessment and therapy monitoring that are uncapable of attending face-to-face visits due to quarantine measures and other personal problems.

In our opinion, telemedicine will become an integral part of daily clinical practice [8]. 

However, according to Borgia et al., teledermatology cannot replace face-to-face visits since the physician–patient relationship is at the basis of therapeutic management. The alternation between teleconsultation and face-to-face visits may be an effective strategy. Thus, dermatologists should keep in mind the possible future role of teledermatology in daily clinical practice. Certainly, new tools and further improvement are required to offer patients a tailored teledermatological approach.

## Data Availability

Data are reported in the current study.
